# The N2-Src neuronal splice variant of C-Src has altered SH3 domain ligand specificity and a higher constitutive activity than N1-Src

**DOI:** 10.1016/j.febslet.2015.05.033

**Published:** 2015-07-08

**Authors:** Sarah Keenan, Philip A. Lewis, Sarah J. Wetherill, Christopher J.R. Dunning, Gareth J.O. Evans

**Affiliations:** Department of Biology and Hull York Medical School, University of York, Wentworth Way, York YO10 5DD, UK

**Keywords:** DMEM, Dulbecco’s minimal Eagle’s medium, NMDA, N-methyl d-aspartate, PTP1B, protein tyrosine phosphatase 1B, SFK, Src family kinase, SH2, Src homology 2, SH3, Src homology 3, Src, Tyrosine protein kinase, Kinase assay, Enzyme kinetics, Src homology 3 domain, Splice variant

## Abstract

N2-Src is a poorly understood neuronal splice variant of the ubiquitous C-Src tyrosine kinase, containing a 17 amino acid insert in its Src homology 3 (SH3) domain. To characterise the properties of N2-Src we directly compared its SH3 domain specificity and kinase activity with C- and N1-Src in vitro. N2- and N1-Src had a similar low affinity for the phosphorylation of substrates containing canonical C-Src SH3 ligands and synaptophysin, an established neuronal substrate for C-Src. N2-Src also had a higher basal kinase activity than N1- and C-Src in vitro and in cells, which could be explained by weakened intramolecular interactions. Therefore, N2-Src is a highly active kinase that is likely to phosphorylate alternative substrates to C-Src in the brain.

## Highlights

• N2-Src is a previously uncharacterised neuronal splice variant of C-Src kinase.
• Tyrosine phosphorylation by C-Src is enhanced by SH3 peptide ligands.
• Ideal C-Src SH3 ligands do not enhance substrate phosphorylation by N2- or N1-Src kinase.
• N2-Src is more active than C- and N1-Src in vitro and in cells.
• N2-Src is likely to have alternative substrates in the brain.

## 1 Introduction

**Fig. 1:**
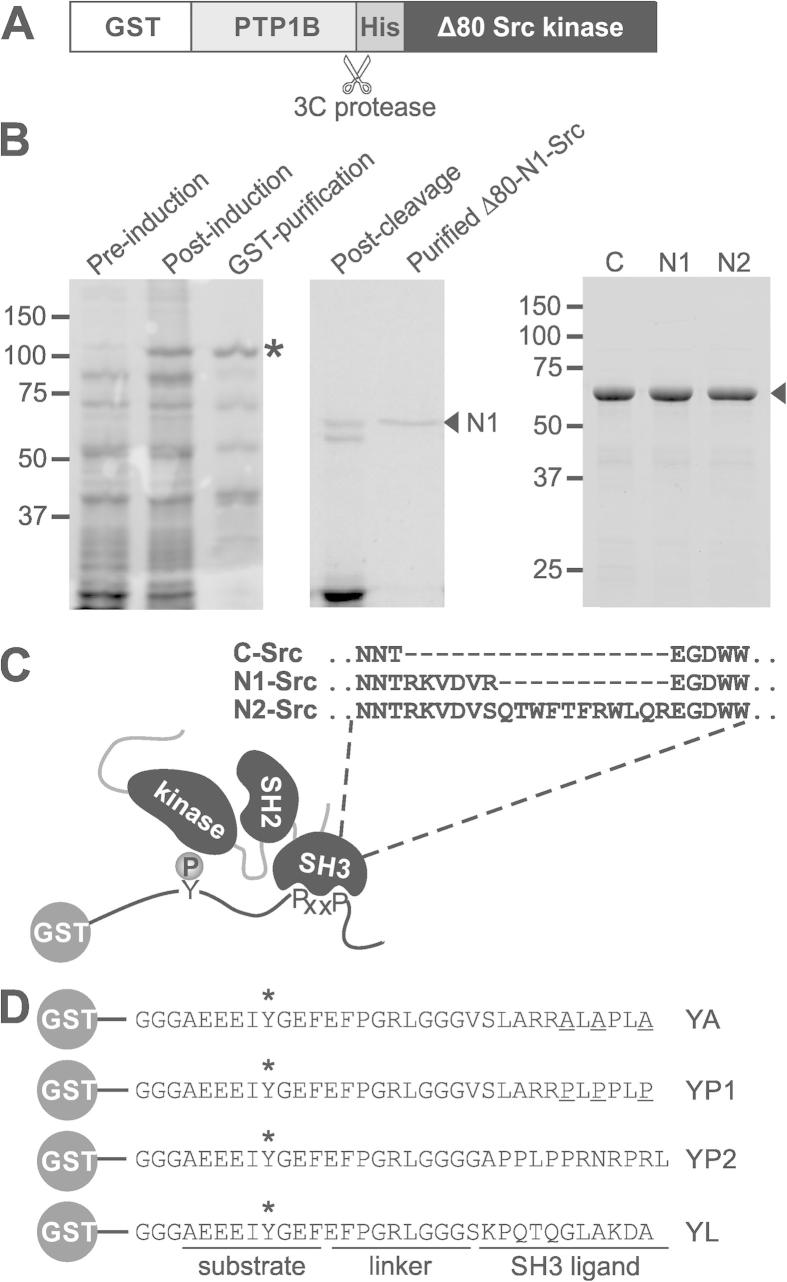
Expression and purification of active Src kinases for in vitro kinase assays. (A) Schematic of the GST-PTP1B-His-Δ80Src construct (lacking the first 80 residues of rat full length Src) used to express active Src kinases in *E. coli*. The 3C protease site to produce His-Src is indicated. (B) Coomassie stained SDS–PAGE gels showing the stages of a representative expression and purification of GST-PTP1B-His-Δ80N1-Src (asterisk; left panel) and its cleavage to yield His-Δ80N1-Src (arrow; middle panel). The right panel shows a comparison of purified His-Δ80C-, N1- and N2-Srcs (arrow). (C) Schematic showing the principles of an in vitro Src kinase assay in which GST-peptide substrates contain a tyrosine residue that can be phosphorylated by the Src kinase domain, and a proline rich sequence inserted at the position of PxxP to assess the effect of SH3 domain binding. The primary sequences of the n-Src loops of the C-, N1- and N2-Src SH3 domains are also depicted. (D) Primary sequences of the GST-peptide substrates used in this study. All substrates contain a phosphorylatable tyrosine residue (*). YP1 and YP2 contain canonical Class I and Class II C-Src SH3 domain binding sequences. YA is a mutant of YP1 in which the prolines have been substituted with alanine (underlined) and YL contains the SH2:kinase linker sequence.

The Src family of non-receptor tyrosine kinases (SFK) play fundamental roles in growth factor receptor and cell adhesion signalling in diverse processes including proliferation, differentiation, migration, membrane trafficking and electrical excitability [1]. Of the 11 SFK members, C-Src, Fyn, Yes, Lck and Lyn are highly enriched in the mammalian brain and regulate major functions, including neuronal differentiation, axon guidance, neurotransmitter release and learning and memory [2–5]. C-Src has two splice variants expressed solely in neuronal tissue [6–8], termed N1- and N2-Src, which arise from insertions of the mini-exons N1 or N1 & N2, respectively. N1-Src contains a six amino acid insert in the n-src loop of its Src homology 3 (SH3) domain, while in N2-Src, the N1 and N2 mini-exons insert a total of 17 amino acids (Fig 1C; [7,9]). Neuronal splicing of C-Src and the charge distribution of the N1-Src inserts is conserved from teleost fish to man [10–13], while N2-Src is only found in mammals, suggesting it is important for higher brain functions. However, the regulation and in vivo substrates of the neuronal Srcs remain largely unknown.

Due to the critical role played by SH3 domains in substrate recognition, it was previously predicted that N-Src binding to known C-Src ligands is altered [14]. Indeed, studies have shown that interactions between the N1-Src SH3 domain and a number of neuronal proteins are reduced or abolished compared to C-Src [15–19]. Although there are several putative N1-Src SH3 domain binding partners [20–23], none of these interactions have been confirmed in vivo and only the NMDA (N-methyl d-aspartate) receptor subunit, NR2A, has been shown to be phosphorylated by N1-Src (in vitro; [24]). To date, no binding partners or substrates have been identified for N2-Src, however, high levels of N2-Src expression correlate with a good prognosis in the childhood cancer neuroblastoma [25].

In addition to altered substrate binding, the N1-Src SH3 domain has been shown to increase its kinase activity in cells above that of C-Src [8,26,27]. We hypothesise these observations are due to a reduction in the ability of the N1-Src SH3 domain to form intramolecular association with the Src homology 2 (SH2):kinase linker that normally constrains the activity of C-Src [28,29]. In this study we performed the first biochemical investigation of N2-Src kinase since it was cloned in 1990 [7]. We show that similar to N1-Src, N2-Src has a low affinity for substrates containing canonical C-Src SH3 ligands. We also observed that N1-Src, and in particular N2-Src, have a lower threshold for activation by auto-phosphorylation, likely due to weaker intramolecular interactions between the SH3 domain and the SH2:kinase linker.

## 2 Materials and methods

### 2.1 Materials

Mouse monoclonal anti-phosphotyrosine (clone PY20) was obtained from BD Bioscience (Oxford, UK). Rabbit polyclonal anti-Src pY527 and anti-Src pY416 were from Cell Signaling Technologies (Hitchin, UK). Mouse anti-β-actin was from Proteintech (Manchester, UK). Mouse anti-FLAG (clone M2), anti-mouse and anti-rabbit HRP-conjugated secondary antibodies and 2X SDS sample buffer were from Sigma (Poole, UK). Unless stated otherwise, all other reagents were from Sigma.

### 2.2 Plasmids

The C-, N1- and N2-Src kinases and SH3 domains were cloned by PCR from rat brain cDNA (Clontech, Oxford, UK). Plasmids for expressing recombinant active Δ80C-Src, N1- or N2-Src in *Escherichia coli* were prepared by inserting cDNA encoding His-tagged rat Srcs lacking the first 80 amino acids into pGEX4T-1 (GE Healthcare, Little Chalfont, UK) containing the human protein tyrosine phosphatase 1B (PTP1B) catalytic domain with a 3C protease site at its C-terminus (Fig. 1A). FLAG-tagged Src mammalian expression plasmids were prepared by sub-cloning full length Src cDNA into pFLAG-N1 (pFLAG-N1 was created by replacing the GFP sequence with a FLAG tag in pEGFP-N1; Clontech), in which a GSGS linker was introduced at the C-terminus prior to the tag. The GSGS linker has been previously used to prepare C-Src-GFP and does not affect C-Src activity [30]. Plasmids comprising a Src substrate and SH3 ligand fused to GST (YA, YP1 and YP2) were prepared by ligating annealed oligonucleotides encoding an ideal Src family kinase substrate (AEEEIYGEF; [31]) into pGEX6P-1 (GE Healthcare; 5′ BamHI and 3′ EcoRI sites) and then SH3 docking sequences (Class I, YP1; VSLARRPLPPLP and Class II, YP2; PPLPPRNRPRL; [32]) were ligated into 3′ SalI and 5′ NotI sites (Fig. 1D). A plasmid encoding the cytoplasmic tail of synaptophysin fused to GST (pGEX-Syp-C-term) was a kind gift from Prof Michael Cousin (University of Edinburgh).

### 2.3 Protein purification

Recombinant His- and GST-fusion proteins were expressed and purified according to a previously described protocol with minor modifications for Src kinase expression [33]. For the production of active Src kinases, BL21 *E. coli* cultures at an O.D_600_ = 1 were induced with 0.5 mM IPTG and incubated at 18 °C overnight, shaking at 250 rpm. Following purification with glutathione resin (Genscript), the His-tagged kinases were cleaved from GST-PTP1B by incubation with PreScission 3C protease (GE Healthcare) at 4 °C overnight. The purified kinases were diluted 1:1 in kinase storage buffer (50 mM Tris–HCl, pH 7.5, 10 mM NaCl, 0.05 mM EDTA, 1 mM DTT, 10% glycerol, 1 mg/ml BSA, 0.05% NP-40), and stored at −80 °C. Approximately 0.5 mg pure kinase was obtained from a 1 l culture of *E. coli*.

### 2.4 In vitro kinase assays

Phosphorylation reactions were prepared in kinase reaction buffer (100 mM Tris, 10 mM MgCl_2_, pH 7.5) and initiated by the addition of pre-warmed ATP (0.5 mM final concentration) and incubated at 30 °C for the indicated times. Kinase and substrate concentrations were varied as indicated in the figure legends. Assays were terminated by transfer to ice and the immediate addition of 2X SDS sample buffer. Twenty percent of the reaction was separated on a 15% SDS–PAGE gel and transferred to PVDF membrane (Millipore, Croxley Green, UK). Substrate phosphotyrosine content was determined by immunoblotting with mouse anti-phosphotyrosine (1:1000) and anti-mouse-HRP (1:5000). Immunoreactivity was visualised by incubation of immunoblots with enhanced chemiluminescence reagent (Millipore) and exposure to autoradiography film (Santa Cruz, Dallas, TA). To ensure equal protein loading, samples were stained with Coomassie gel stain to detect the substrates. Densitometric analysis of protein bands on immunoblots was performed with ImageJ [34].

To measure enzyme kinetics, the samples representing each substrate concentration phosphorylated by C-, N1- and N2-Src kinases were separated on a single gel. To normalise densitometry across multiple gels and produce curves across the full concentration range, a standard phosphorylated protein sample (8.3 μM YA phosphorylated by C-Src) was included on each gel. The highest value obtained by densitometry analysis of each curve was set to 1 and all other values were normalised accordingly. Due to the non-quantitative measurement of *V*, *V*_max_ was not determined. *K*_m_ was calculated from three independent experiments by fitting to Michaelis–Menton equations in the enzyme kinetics module of SigmaPlot (Systat, Chicago, IL). Where appropriate, statistical analyses of the data were performed with SigmaPlot software using one- or two-way ANOVA with post hoc pairwise comparisons by Tukey tests.

### 2.5 Cell culture, transfection and detection of Src expression

B104 rat neuroblastoma cells were cultured in DMEM (Dulbecco’s minimal Eagle’s medium; Invitrogen, Paisley, UK) supplemented with 10% foetal calf serum and 1% penicillin/streptomycin (Invitrogen). For the analysis of Src-FLAG expression and phosphorylation by immunoblotting, 3 × 10^4^ cells were plated per well of a 24 well plate and transfected 24 h after plating at a ratio of 1 μg plasmid cDNA:2 μl EcoTransfect reagent according to the manufacturer’s instructions (Oz Biosciences, Marseille, France). Cells were lysed 48 h after transfection in SDS sample buffer and analysed by immunoblotting with anti-FLAG (1:1000), anti-Src-pY416 (1:1000), anti-Src-pY527 (1:1000), anti-phosphotyrosine (1:1000), anti-β-actin (loading control; 1:50 000) and appropriate HRP-conjugated secondary antibodies (1:5000).

## 3 Results and discussion

### 3.1 An in vitro kinase assay for SH3 domain substrate specificity

To perform a direct comparison between the substrate specificities of C-, N1- and N2-Src, we developed an in vitro kinase assay using recombinant truncated Src kinases (lacking the first 80 amino acids) to phosphorylate peptide substrates fused to the C-terminus of GST. The expression of active Src kinases in *E. coli* is problematic, due to the toxicity of tyrosine phosphorylation in bacteria that do not support this form of post-translational modification [35]. To overcome this issue we fused a cleavable tyrosine phosphatase (PTP1B) catalytic domain to the N-terminus of the kinase as previously reported (Fig. 1A; [36]). This modification yielded sufficient pure active kinase to perform in vitro kinase assays (Fig. 1B).

The substrates comprised an ideal Src kinase substrate sequence (AEEEIYGEF; [31], fused to canonical proline rich SH3 ligand sequences (Fig. 1D; YP1 and YP2). A control sequence (YA) was also included in which the prolines of YP1 were substituted with alanine. In all the substrates there were at least ten amino acids between the phosphorylatable tyrosine substrate and the proline rich motif, which was adequate for SH3 domain docking and phosphorylation by Hck and v-Src in a previous study [37].

**Fig. 2:**
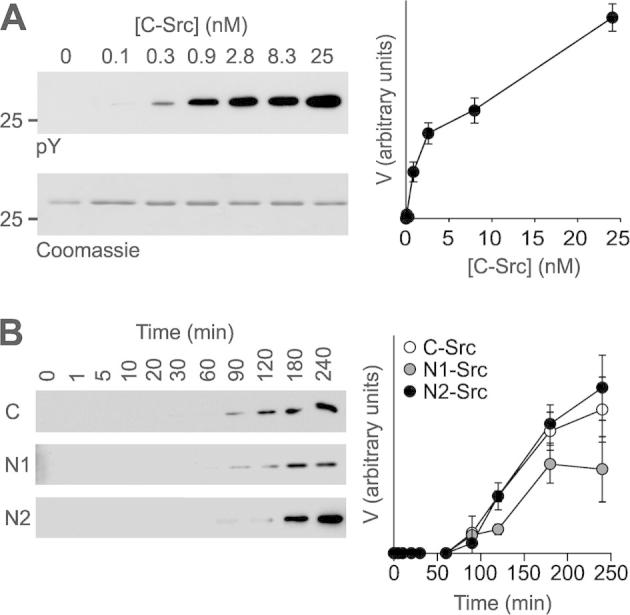
Characterisation of an in vitro kinase assay for Src. All kinase assays were carried out at 30 °C, initiated by the addition of 0.5 mM ATP and halted by transfer to ice and the immediate addition of SDS sample buffer. (A) 25 μM YA (an ideal Src substrate peptide fused to GST, see Fig. 1D) was incubated with the indicated concentrations of His-Δ80C-Src for 90 min. Reactions were separated in duplicate by SDS–PAGE, and either stained with Coomassie (bottom panel) or transferred to PVDF membrane and immunoblotted with an anti-phosphotyrosine antibody (pY; top panel). Values for *V* were obtained from densitometry of the pY immunoreactive bands with ImageJ, normalised to the amount of Coomassie stained YA and plotted in arbitrary units against enzyme concentration. (B) 25 μM YA was incubated with 5 nM C-, N1- or N2-Src for the indicated times and the samples treated and analysed as described in (A). All data are plotted as mean ± S.E.M., from *n* = 3 independent experiments.

**Fig. 3:**
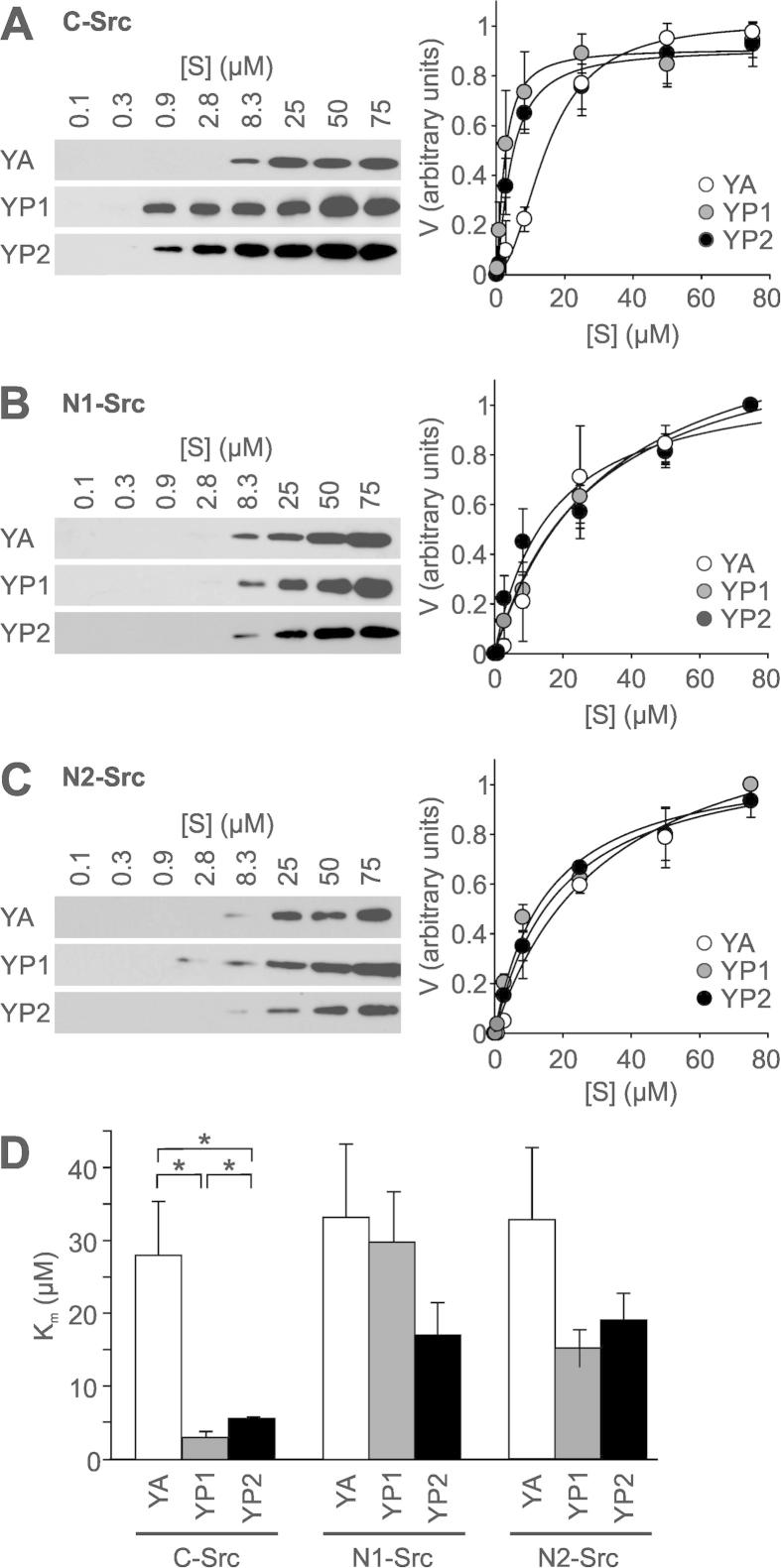
Canonical Class I and Class II proline rich motifs enhance phosphorylation of an ideal substrate by C-Src but not the N-Srcs. Phosphorylation of substrates containing Class I (YP1) or Class II (YP2) proline rich motifs, at the indicated concentrations, by 5 nM HisΔ80C- (A), N1- (B) or N2-Src (C) was performed for 90 min. The initial rate (*V*) for each reaction was calculated by densitometry (by ImageJ) of anti-phosphotyrosine immunoblots (normalised as described in Fig. 1) and plotted against substrate concentration. Curve fitting to calculate Michaelis–Menten parameters was performed using SigmaPlot. (D) Comparison of the *K*_m_ of YA, YP1 and YP2 substrate phosphorylation by C-, N1- and N2-Src. All data are plotted as mean ± S.E.M., from *n* = 3 independent experiments. Statistical significance was determined by two way ANOVA, ^*^P < 0.05.

**Fig. 4:**
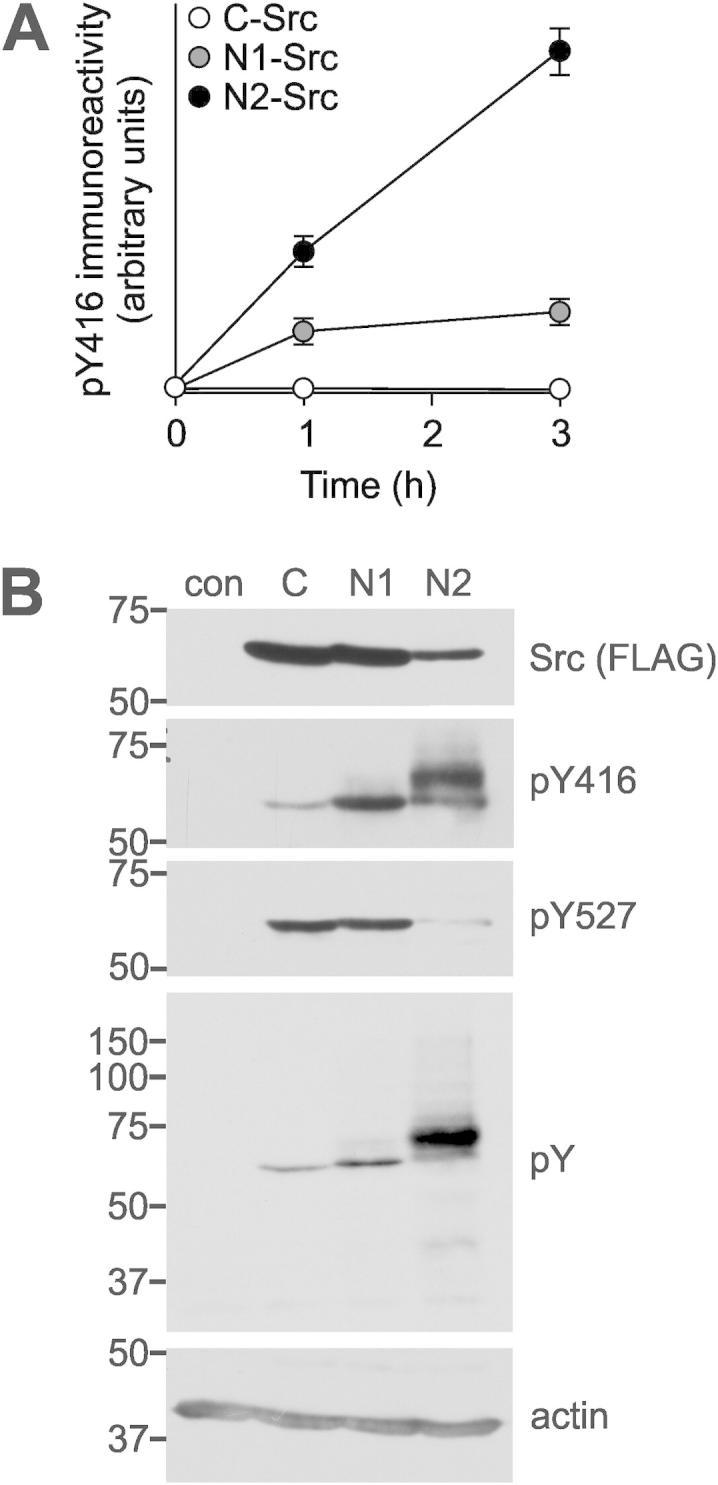
N-Srcs have enhanced levels of Y416 auto-phosphorylation in vitro and in cells compared to C-Src. (A) 200 nM HisΔ80C-, N1- or N2-Src was incubated at 30 °C with 0.5 mM ATP for 0, 1 or 3 h and processed for immunoblotting with anti-pY416 and anti-His (loading control). pY416 immunoreactivity was obtained by densitometry with ImageJ and normalised to the anti-His signal. Data are plotted as mean ± S.E.M., from *n* = 3 independent experiments. (B) B104 cells were transfected with plasmids encoding FLAG-tag alone (control) or C-terminal FLAG-tagged full length C-, N1- or N2-Src, and the lysed after 48 h. The lysates were analysed by immunoblotting with anti-FLAG (FLAG), anti-phosphotyrosine (pY), anti-pY416 (active Src), anti-pY527 (inactive Src) and β-actin (loading control). The immunoblots are representative of at least three independent experiments.

To measure enzyme kinetics, experiments were first conducted with C-Src phosphorylation of YA to establish conditions where phosphate incorporation was linear with time (Fig. 2). The rate of the reaction (*V*) was determined by immunoblotting with an anti-phosphotyrosine antibody, which was quantified by densitometry (Fig. 2). This assay therefore gave arbitrary values for *V*_max_, but enabled numerical calculation of *K*_m_ to assess the affinity of the Src kinases for their substrates. Varying the kinase concentration (Fig. 2A) revealed that a concentration of 5 nM kinase would be suitable for kinetic measurements. A timecourse of YA phosphorylation by C-, N1- or N2-Src revealed a lag in activity until approximately 60–90 min, where phosphorylation of YA then increased rapidly (Fig. 2B). This is consistent with the time taken for kinase auto-phosphorylation (Fig. 4A), which is linked with activation of the catalytic domain. The phosphorylation by all three kinases was equivalent at 90 min and this reaction time was used for subsequent kinetic experiments. Utilising these parameters whilst varying the substrate concentration gave classical Michaelis–Menten kinetics (Fig. 3A), with a *K*_m_ of 28.7 ± 7.4 μM for phosphorylation of the YA substrate by C-Src, which compares favourably with the previously observed *K*_m_ of 33 μM for C-Src phosphorylating the free AEEEIYGEF peptide [31]. When we compared the phosphorylation of the YA substrate by C-, N1- and N2-Src, the *K*_m_s were not significantly different between the three kinases (Fig. 3D).

### 3.2 The N-Src SH3 domains have different ligand specificity to C-Src

To assess whether the N-Src SH3 domain specificity differs from that of C-Src, phosphorylation of the YP1 and YP2 substrates was investigated. In addition to an ideal Src phosphorylation site, YP1 and YP2 contain Class I and Class II PxxP motifs respectively, defined by a positively charged arginine residue before (Class I) or after (Class II) the PxxP motif (Fig. 1D). It was previously shown that peptides encoding an SH3 ligand C-terminal to a tyrosine phosphorylation site lowered the *K*_m_, but did not affect the *V*_max_, of v-Src [37]. The presence of a Class I or Class II (Fig. 3A) ligand C-terminal to the kinase substrate sequence significantly enhanced phosphorylation by C-Src with *K*_m_s of 4.3 ± 1.0 μM and 6.1 ± 1.2 μM for YP1 and YP2 respectively, compared to 28.7 ± 7.4 μM for YA (Fig. 3D).

In stark contrast to C-Src, the *K*_m_s for the phosphorylation of YP1 and YP2 by N1- (Fig. 3B) and N2-Src (Fig. 3C) were not significantly different from YA (Fig. 3D). These are the first data to demonstrate that SH3 domain specificity affects N-Src affinity for a substrate in a kinase assay, and support several immunoprecipitation, pulldown or in vitro binding assays showing differences between the C- and N1-Src SH3 domains. Furthermore, NMR studies of the C-Src SH3 domain have shown that the n-Src loop, the site of N-Src splicing, contacts basic residues up- or downstream of the core PxxP motif of Class I or Class II SH3 ligands respectively [14,38]. These studies provide a structural hypothesis for how splicing alters SH3 specificity, suggesting that specific ligands for the N1- and N2-SH3 domains might have alternative flanking residues outside the PxxP motif.

### 3.3 High auto-phosphorylation of N2-Src in vitro and in cells

Auto-phosphorylation of Y416 (based on the amino acid numbering of chicken C-Src and used from here onwards for C, N1 and N2-Src) is a key step in the activation of the Src kinase domain and it has been previously reported that N1-Src has a higher constitutive activity than C-Src [26,27]. In our in vitro kinase assays we were unable to detect kinase auto-phosphorylation due to the low kinase concentration (5 nM). Assays were therefore performed in which 200 nM C-, N1 or N2-Src was incubated with ATP at 30 °C and samples removed at 0, 1 and 3 h prior to analysis of pY416 immunoreactivity. C-Src phosphorylation was barely detectable at 3 h, even with a long exposure of the immunoblot, whereas phosphorylation of the neuronal Srcs was readily detectable, with N2-Src approximately 4-fold more phosphorylated than N1-Src at 3 h (Fig. 4A). Comparing this timecourse with that of YA in Fig. 2A suggests that in vitro, despite a high auto-phosphorylation activity, phosphorylation of an ideal substrate is not significantly greater. There are other reports where pY416 does not directly correlate with activity, for example, v-Src phosphorylates substrates 10-fold more than C-Src but its auto-phosphorylation is only between 1 and 2-fold higher than C-Src [39].

We next determined the kinases’ phosphorylation status in cells, where they are subject to physiological regulation. In addition to detecting pY416, phosphorylation of Y527 was also analysed. Y527 phosphorylation at the C-terminus of Src by Csk promotes intramolecular association with the SH2 domain and is indicative of the inactive form of the kinase. Cell lysates from B104 rat neuroblastoma cells transfected with FLAG-tagged C-, N1- or N2-Src were analysed by immunoblotting with anti-pY416 and anti-pY527 antibodies. We observed no pY416 signal in untransfected cells and a weak signal in cells transfected with C-Src, however, pY527 immunoreactivity was readily detected for C-Src (Fig. 4B). Therefore, it is likely that C-Src expressed in the cell is predominantly in an inactive conformation as previously reported [40]. In contrast, strong pY416 immunoreactivity was observed in cells transfected with N1- or N2-Src in bands that aligned with those in an anti-FLAG immunoblot (Fig. 4B). An additional intense band was observed migrating above N2-Src, that might represent activation of another endogenous SFK. Surprisingly, N1-Src had a pY527 signal comparable to C-Src while the signal for N2-Src was greatly reduced (Fig. 4B). The neuronal Srcs therefore have a higher basal level of auto-phosphorylation than C-Src, inferring a higher basal level of kinase activity. The enhanced auto-phosphorylation of N1-Src above that of C-Src has been previously reported [8,26,27] and we now show that N2-Src has even greater activity, both in vitro and in cells.

To assess whether transfection of the apparently highly active N-Srcs resulted in increased cellular substrate phosphorylation, we probed the same lysates for total phosphotyrosine content. This revealed that the major phosphorylated proteins within the cells are the neuronal Srcs themselves (Fig. 4B), presumably at pY416 and pY527. In addition, readily detectable phosphotyrosine immunoreactivity of other cellular proteins was observed for N2-Src transfected cells and faint bands were observed for N1-Src. Thus, the extent of basal phosphorylation of substrate proteins in cells for C-, N1- and N2-Src correlates with their auto-phosphorylation activity in cells and perhaps we would observe a similar correlation in vitro with *bona fide* N-Src substrates.

### 3.4 Reduced affinity of the N-Srcs for a SH2:kinase linker peptide

**Fig. 5:**
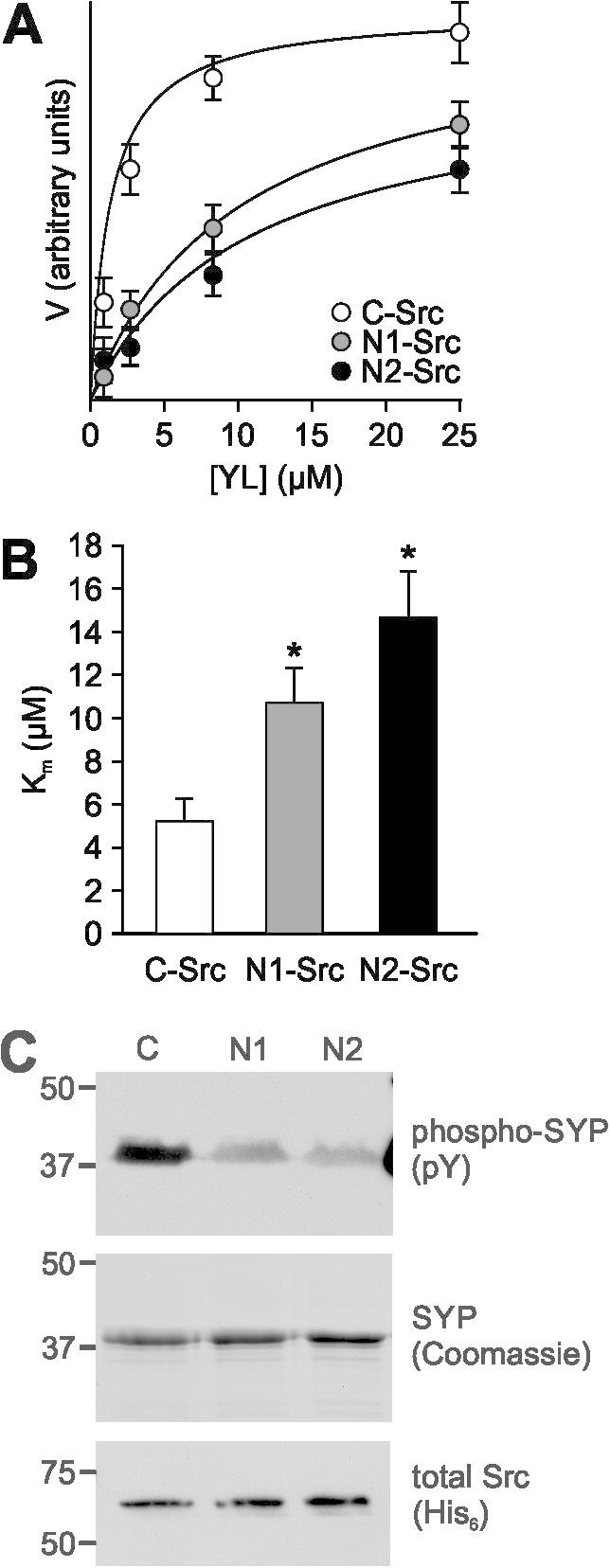
The Src SH2:kinase linker sequence and the synaptic vesicle protein synaptophysin are poor substrates for the N-Srcs compared to C-Src. Phosphorylation of a substrate containing the Src SH2:kinase linker, at the indicated concentrations, by 5 nM HisΔ80C-, N1- or N2-Src was performed for 90 min. (A) The initial rate (*V*) for each reaction was calculated by densitometry (by ImageJ) of anti-phosphotyrosine immunoblots and plotted against substrate concentration. Curve fitting to generate Michaelis–Menten parameters was performed using the kinetics module in SigmaPlot. (B) Comparison of the *K*_m_ of substrate phosphorylation. All data are plotted as mean ± S.E.M., from *n* = 3 independent experiments. Statistical significance was determined by one way ANOVA, ^*^P < 0.05. (C) 10 μM GST-synaptophysin C-terminus (SYP) was incubated at 30 °C with 66 nM HisΔ80C-, N1- or N2-Src and 0.5 mM ATP for 3 h. Reactions were separated in duplicate by SDS–PAGE, and either stained with Coomassie (SYP) or transferred to PVDF membrane and immunoblotted with anti-phosphotyrosine (pY) and anti-His (His_6_). The images are representative of at least three independent experiments.

The enhanced auto-phosphorylation activity of the N-Srcs is reminiscent of mutants of C-Src that reduce intramolecular interactions. The availability of Y416 in the kinase domain activation loop is tightly regulated by two intramolecular associations: SH2 binding to a C-terminal phosphotyrosine (pY527) and SH3 binding to the SH2:kinase linker. Evidence is accumulating to suggest that the disruption of *either* of these interactions, akin to an ‘OR-gate’ switch, is sufficient to permit auto-phosphorylation on Y416 for full activity [29,41]. We hypothesised that the increased cellular basal activity of the N-Srcs was due to a perturbation of the intramolecular interaction between the SH3 domain and the SH2:kinase linker, which normally constrains SFK kinase activity. The function of the linker (^248^SKPQTQGLAKDA) was first delineated by deletions and mutations in the SH3 domains of Src and Hck that increased kinase activity [42,43]. To investigate the affinity of the N-Src SH3 domains for the linker sequence, we assayed the phosphorylation of a GST-peptide substrate (Fig. 5A), in which the linker sequence was inserted next to the ideal substrate motif (YL; Fig. 1D). The *K*_m_ for C-Src phosphorylation of YL was 5.2 ± 1.1 μM (Fig. 5B), similar to the *K*_m_ for the phosphorylation of the ideal substrates YP1 and YP2 (Fig. 3D). This was somewhat surprising, considering the linker does not contain a canonical PxxP motif. The *K*_m_ for YL phosphorylation by both N1- and N2-Src was significantly decreased to 10.7 ± 1.5 and 14.6 ± 2.1 μM respectively (Fig. 5B). This implies that the N-Src SH3 domains bind more weakly to the linker sequence, due to steric hindrance by the n-Src loop. In the context of the full length kinase, a reduction in this intramolecular interaction could explain why these kinases are more susceptible to auto-phosphorylation.

### 3.5 Differential specificity of the Srcs for a neuronal C-Src substrate

Having observed differential specificity of the N-Srcs for ideal SH3 ligands, we investigated the phosphorylation of an established in vivo C-Src substrate in brain, synaptophysin, a synaptic vesicle membrane tyrosine phosphoprotein [44,45]. We confirmed that the cytoplasmic C-terminus of synaptophysin is an excellent substrate for C-Src (Fig. 5C), but surprisingly observed little phosphorylation of the protein by N1- and N2-Src. This experiment reaffirms that SH3 domain specificity overrides the auto-phosphorylation activity of the N-Srcs for determining the extent of phosphorylation of their substrates.

Taken together, we have shown that in cells and in vitro*,* N2-Src has a high auto-phosphorylation activity, likely driven by weak intramolecular interactions. The N-Src SH3s have a weak affinity for peptide substrates containing Class I and Class II C-Src SH3 ligands and future work is required to establish the in vivo substrates of the N-Srcs and the structural basis of high affinity binding to the N-Src SH3 domains. Such studies could facilitate the development of isoform-specific inhibitors that can distinguish the kinases by disrupting SH3 ligand binding.
